# Fatigue and Mental Status of Caregivers of Severely Chronically Ill Patients

**DOI:** 10.1155/2020/6372857

**Published:** 2020-09-07

**Authors:** Sung-Goo Kang, Sang-Wook Song, Se-Hong Kim, Yi-Jin Kang, Young-Rye Kim, Youngmi Eun

**Affiliations:** ^1^Department of Family Medicine, St. Vincent's Hospital, College of Medicine, The Catholic University of Korea, Seoul, Republic of Korea; ^2^Department of Nursing, St. Vincent's Hospital, College of Medicine, The Catholic University of Korea, Seoul, Republic of Korea; ^3^Department of Family Medicine, Yeouido St.Mary's Hospital, College of Medicine, The Catholic University of Korea, Seoul, Republic of Korea

## Abstract

**Background and Aims:**

Fatigue is an unpleasant experience accompanied by functional deterioration involving both mental and physical factors. Caregivers of patients with severe illnesses who require long-term treatment often experience marked physical and mental fatigue. This study investigated the factors affecting fatigue among caregivers of patients with severe chronic diseases.

**Methods:**

The study enrolled 100 caregivers of patients providing home care nursing at a university hospital in Gyeonggi-do of Korea, including 47 caregivers caring for cancer patients and 53 caregivers caring for chronic disease patients (e.g., dementia, amyotrophic lateral sclerosis, and Parkinson's disease). The degree of fatigue was measured using the Korean version of the multidimensional fatigue inventory (MFI-K). Caregiver depression and anxiety were examined using the Hospital Anxiety and Depression Scale.

**Results:**

The average MFI-K score of all caregivers was 60.43 ± 13.77 and did not differ significantly between those caring for cancer patients and those caring for patients with severe chronic diseases (62.15 ± 13.27 vs. 58.49 ± 14.20, respectively, *p*=0.186). The longer the disease duration, the greater the general and physical fatigue of the caregiver (*r* = 0.284, *p*=0.004). However, caregiver mental fatigue did not differ according to disease duration (*r* = 0.169, *p*=0.094). The main factors affecting caregiver general and physical fatigue were caregiver anxiety and depression and patient's disease duration.

**Conclusions:**

The caregivers of patients with cancer or chronic severe illnesses experience high levels of fatigue: the longer the disease duration, the greater the degrees of depression, anxiety, and physical fatigue experienced by the caregivers. Such caregivers need strategies to manage their fatigue and depression.

## 1. Introduction

Fatigue is caused by a variety of factors and is often encountered in primary care settings. Fatigue is generally defined as overwhelming, persistent mental or physical fatigue, weakness, and exhaustion [1]. While the cause of fatigue is often unclear, fatigue is affected by mental factors such as daily stress, anxiety, and depression [2] and by physical factors such as cancer, cardiovascular disease, and infection [3, 4]. General fatigue improves with rest and usually does not interfere with the daily activities. However, pathologic fatigue may persist for >6 months, reduces quality of life, negatively affects emotional and social functions, and can impair daily life [4]. Chronic fatigue does not improve with rest. Therefore, the management and evaluation of fatigue is necessary.

As society ages, more patients are requiring home care for chronic diseases, which cause emotional disability and economic loss to patients and their families [5]. The family care process causes physical, mental, psychological, and economic problems and is perceived as burdensome [6]. For example, relatives acting as family caregivers have a higher mortality rate than that of noncaregiver relatives [5]. Therefore, understanding and managing the emotional and physical conditions of caregivers is important.

While some studies have examined caregivers, few have examined caregiver fatigue in home care nursing, and the fatigue was assessed using only simple tools [7]. Recently, a reliable multidimensional fatigue inventory (MFI) scale with proven reliability and validity was adapted for use in Korea (MFI-K) [8]. Therefore, this study examined fatigue and associated factors using the MFI scale in caregivers who care for patients with chronic disease or cancer at home.

## 2. Methods

### 2.1. Subjects

This study examined caregivers for patients with terminal cancer (e.g., stomach, lung, pancreatic, colon, and bladder cancers) or chronic diseases (e.g., dementia, amyotrophic lateral sclerosis, and Parkinson's disease) from June 2019 to January 2020. These caregivers provided home care nursing at a university hospital in Gyeonggi-do, Korea. The subjects were adults between the ages of 20 and 75 years and excluded caregivers with diseases that affect fatigue or mental disabilities.

The caregivers' gender, age, cohabitation status, height, weight, underlying diseases, alcohol consumption (more than once a week or less), smoking history, and education level were investigated to identify caregiver conditions that could affect fatigue. The patients' age, diagnosis, disease duration, and morbidities were also recorded.

This study was conducted in compliance with the ethics and safety guidelines of the institutional review board (approval no. VC19QESI0112), and all caregiver subjects provided informed consent.

### 2.2. Multidimensional Fatigue Inventory-Korean Version (MFI-K)

Fatigue can be evaluated using one-dimensional instruments such as a visual analogue scale or the fatigue severity scale developed by Krupp [9, 10]. MFI is one of the most useful multidimensional tools used for fatigue research. MFI was developed by Smets et al. and consists of five subscales: general fatigue, physical fatigue, reduced activity, reduced motivation, and mental fatigue. MFI has been translated into English, French, and Swedish and validated in each language [11–13].

Kang et al. developed and validated a Korean version [8]. Unlike the English version, MFI-K has four subscales: general and physical fatigue, mental fatigue, reduced activity, and motivation. Each subscale includes six (general and physical fatigue and mental fatigue) and four items (reduced activity and motivation) with five-point Likert scales. The total scores of MFI-K range from 20 to 100, with higher scores indicating greater fatigue. In a previous Korean study, the average of the total MFI-K scores for no or mild fatigue, moderate fatigue, and severe fatigue was 41.36 ± 10.52, 52.85 ± 9.63, and 61.23 ± 10.54, respectively [8].

### 2.3. Epworth Sleepiness Scale (ESS)

The ESS is a self-assessment tool used to evaluate daytime sleep and sleepiness and is composed of eight questions answered using a Likert scale, with the score for each question ranging from 0 to 3 and the total score ranging from 0 to 24. Higher ESS scores indicate more daytime sleepiness, and a score >10 points indicates excessive daytime sleepiness [14, 15]. The Korean version of the ESS has high reliability with Cronbach's *α* = 0.90 [16].

### 2.4. Hospital Anxiety and Depression Scale (HADS)

Zigmond and Snaith [17] developed the HADS self‐assessment instrument for detecting depression and anxiety in the outpatient clinic setting. Oh and Min translated the HADS into the Korean language and established its validity and reliability [18]. The HADS consists of 14 questions; the seven odd-numbered questions are an anxiety subscale and the seven even-numbered questions are a depression subscale. Each item is scored on a scale of 0–3 with each subscale score ranging from 0 to 21 (normal range, 0–7; possible presence of a mood disorder, 8–10; probable presence of a mood disorder, 11–21). This test is a simple, time-saving screening test that can readily identify patients who require psychiatric intervention.

### 2.5. Statistical Analysis

To compare the general characteristics between caregivers of patients with terminal cancer and those of chronically ill patients, the mean and standard deviation values of continuous variables were compared using independent *t*-tests and the frequencies and percentages of categorical variables were compared using chi-square or Fisher's exact tests. Pearson's correlation analysis was used to assess the correlations between the MFI-K score and other factors. Multiple regression analysis was used to identify factors associated with the MFI-K total score, general and physical fatigue score, and mental fatigue score. The data were analyzed using SPSS ver. 16.0 (SPSS, Chicago, IL, USA). Two-sided *p* < 0.05 was considered statistically significant.

## 3. Results

### 3.1. Participants

The study enrolled 100 subjects: 53 caregivers of chronically ill patients and 47 caregivers of terminal cancer patients. Of the participants, 72% were women and 97% were living with the patients. Comparing the characteristics between caregivers of chronically ill patients and those of terminal cancer patients, only the duration of the patient's disease differed significantly (*p* < 0.001); the MFI-K, ESS, and HADS scores did not differ (Table 1).

### 3.2. The Correlation between MFI-K and Other Factors

The MFI-K total, general and physical fatigue, and mental fatigue scores were all positively correlated with the ESS total score, HADS-anxiety score, HADS-depression score, and MFI-K score (Table 2). There were positive correlations between the MFI-K total score and patient age, between the MFI-K general and physical fatigue score and disease duration, and between the MFI-K mental fatigue score and caregiver age.

### 3.3. Factors Associated with Caregiver MFI-K Total, General and Physical Fatigue, and Mental Fatigue Scores

Using regression analysis, the caregiver MFI-K total and mental fatigue scores increased with caregiver age, HADS-depression score, and ESS total score (Tables 3 and 4) (*p* < 0.05). The caregiver MFI-K general and physical fatigue score was associated with patient's disease duration and caregiver HADS anxiety and depression scores (Table 5) (*p* < 0.05).

## 4. Discussion

This study examined fatigue in caregivers of terminal cancer patients and those of chronically ill patients using multidimensional instruments. The overall fatigue and mental fatigue scores increased when the caregiver was old and the daytime sleepiness or depression was severe. The physical fatigue of the caregivers also increased with the patient's disease duration and with caregiver depression or anxiety.

In this study, the degree of fatigue and depression, anxiety, and insomnia did not differ significantly between the caregivers of terminal cancer patients and those of chronically ill patients. However, the average total MFI-K scores were 58.49 and 62.15 for the caregivers of terminal cancer and those of chronically ill patients, respectively, indicating moderate to severe fatigue. By comparison, in a study of caregivers of patients without disease in Korea, the average MFI-K total scores of caregivers with moderate and severe fatigue was 52.85 and 61.23, respectively [8]. Using a visual analogue scale for fatigue (18 items, two-dimensional scale), another study compared the fatigue levels of caregivers of patients with cancer, Alzheimer's disease, and Parkinson's disease with those of a control group and found that the caregivers had more sleep difficulties and fatigue than did the control group. However, there were no significant differences according to the patient's illness [7]. In a study of caregivers of the elderly with disabilities, one-third of the caregivers had moderate fatigue, and 40% had scores indicating depression [19].

HADS-depression and HADS-anxiety scores ≥11 suggest the presence of mood disorders, and scores of 8–10 are borderline abnormal. In our caregivers, the average HADS-depression score was more than 10.45, suggesting a high risk of depressive mood [20]. Caregivers may experience excessive fatigue and depression from their continuous and demanding work. Therefore, in addition to managing care for the patients, the medical staff need to manage the fatigue and depression of caregivers.

We found that caregiver age, daytime sleepiness, depression, and anxiety and the patient's duration of illness potentially affect the caregiver's fatigue. Although there are few studies of caregiver fatigue, the effect of caregiver age on fatigue varies. A study of the families of patients undergoing palliative care found that younger relatives had greater mental fatigue and participated in fewer activities compared with older relatives and sleepiness was not related to fatigue [21]. Another study that evaluated the caregivers of cancer patients found that age, economic level, and care duration were not related to the degree of caregiver fatigue [22]. However, those studies were limited by the small numbers of subjects and by the shorter duration of the caregivers' help.

In a large population study conducted in Denmark, compared with 20-year-old women, the fatigue was less with age, but the general fatigue, physical fatigue, and reduced activity scores were higher in the persons with physical illness, and the reduced activity score was higher in the persons with depression [23]. In this study, the average caregiver age was over 60 years, 40% had an underlying disease, and the HADS-depression score was high. These factors may affect the relationship between age and fatigue in this study. Thus, further prospective research adjusted for associated factors is needed to determine the causal relationship between age and fatigue in caregivers.

Our study is meaningful in that the study evaluated factors affecting fatigue in the caregivers of severely chronically ill patients receiving home care using a complex questionnaire tool. Nevertheless, the study has some limitations. First, this was a cross-sectional study and therefore could not determine causal relationships. Second, there was no control group, but a previous study conducted in Koreans [8] examined the level of fatigue in the general public, and those results could be indirectly compared with the degree of fatigue in our study subjects. Last, the number of participants was limited. Evaluating more factors affecting fatigue in a greater number of subjects is necessary in future studies.

With our aging society, caregiving is emerging as an important social problem. Despite the relatively easy access to medical services in Korea, caregivers still show high levels of fatigue and depression and do not receive proper medical attention. This study found that caregiver's fatigue worsened with increasing caregiver age, depression, anxiety, and sleepiness, as well as with the prolonged duration of the patient's disease. Future prospective studies need to examine how to reduce the fatigue and depressive symptoms of those who care for patients with diseases not likely to improve. In addition, it may be necessary to assess whether treatment of depression and anxiety can improve fatigue, at least in a mental component.

## Figures and Tables

**Table 1:** General characteristics of study participants.

	Patient disease: severe chronic disease	Patient disease: advanced cancer	*p* values
Sex			0.824
Male	14 (26.4)	14 (29.8)	
Female	39 (73.6)	33 (70.2)	
Caregiver age (years)	62.43 ± 14.11	61.40 ± 13.09	0.707
Patient age (years)	68.57 ± 19.04	70.21 ± 15.69	0.641
Patient's disease duration (months)	103.28 ± 84.18	40.13 ± 64.37	<0.001
BMI (kg/m^2^)	24.45 ± 3.87	23.50 ± 3.01	0.181
Sleep duration (hours)	5.66 ± 1.57	5.96 ± 1.44	0.329
ESS total score	7.23 ± 4.58	7.00 ± 4.14	0.797
HADS-anxiety score	8.51 ± 3.94	9.13 ± 5.44	0.514
HADS-depression score	10.45 ± 4.24	10.47 ± 5.03	0.987
Exercise			0.629
No exercise	31 (58.5)	23 (48.9)	
1–3 times/week	7 (13.2)	8 (17.0)	
>3 times/week	15 (28.3)	16 (34.0)	
Smoking			0.184
Nonsmoker	43 (81.1)	35 (74.5)	
Ex-smoker	9 (17.0)	7 (14.9)	
Current smoker	1 (1.9)	5 (10.6)	
Alcohol consumption			0.194
No alcohol	40 (75.)	29 (61.7)	
**>**1 time/week	13 (24.5)	18 (38.3)	
MFI-K total scores	62.15 ± 13.27	58.49 ± 14.20	0.186
General/physical fatigue	22.02 ± 4.15	20.21 ± 5.11	0.057
Mental fatigue	19.62 ± 5.14	16.36 ± 5.49	0.598
Reduced activities	10.45 ± 3.82	10.04 ± 3.41	0.574
Motivation	12.75 ± 3.14	11.87 ± 3.87	0.212

Data are mean ± SD or N (%). *p* values were obtained by independent *t*-tests or chi-square tests or Fisher's exact tests. BMI, body mass index; MFI-K, multidimensional fatigue inventory-Korean version; ESS, Epworth Sleepiness Scale; HADS, Hospital Anxiety Depression Scale.

**Table 2:** Correlation between MFI-K and other factors.

	MFI-K total scores		General/physical fatigue		Mental fatigue	
	*r*	*p*	*r*	*p*	*r*	*p*
Caregiver age	0.049	0.631	0.185	0.065	0.346	<0.001
Patient age	0.436	<0.001	−0.171	0.089	0.100	0.323
Patient's disease duration	0.193	0.055	0.284	0.004	0.169	0.094
BMI	−0.127	0.207	−0.074	0.465	−0.076	0.452
Sleep duration	−0.026	0.794	−0.171	0.090	0.028	0.782
ESS total scores	0.346	<0.001	0.270	0.007	0.431	<0.001
HADS-anxiety score	0.509	<0.001	0.547	<0.001	0.489	<0.001
HADS-depression score	0.576	<0.001	0.529	<0.001	0.526	<0.001
MFI-K total scores	1		0.768	<0.001	0.881	<0.001
General/physical fatigue	0.768	<0.001	1		0.563	<0.001
Mental fatigue	0.881	<0.001	0.563	<0.001	1	
Reduced activities	0.719	<0.001	0.327	0.001	0.514	<0.001
Motivation	0.829	<0.001	0.489	<0.001	0.665	<0.001

*p* values were obtained by Pearson's correlation analysis.

**Table 3:** The factors associated with the caregiver MFI-K total score.

	*β*	*p* value
HADS-depression score	1.270	<0.001
Caregiver age	0.314	<0.001
ESS total score	0.724	0.004

Adjusted for caregiver BMI, sleep duration, HADS anxiety, patient age, and patient's disease duration.

**Table 4:** The factors associated with caregiver MFI-K mental fatigue.

	*β*	*p* value
HADS-depression score	0.424	<0.001
ESS total score	0.401	<0.001
Caregiver's age	0.092	0.005

Adjusted for caregiver BMI, sleep duration, HADS anxiety, patient age, and patient's disease duration.

**Table 5:** The factors associated with caregiver MFI-K general/physical fatigue.

	*β*	*p* value
HADS-anxiety score	0.356	0.002
Patient's disease duration	0.015	0.001
HADS-depression score	0.264	0.025

Adjusted for caregiver age, BMI, sleep duration, HADS anxiety, and patient age.

## Data Availability

The datasets generated and/or analyzed during the current study are available from the corresponding author on reasonable request.
